# Mg^2+^ limitation leads to a decrease in chlorophyll, resulting in an unbalanced photosynthetic apparatus in the cyanobacterium *Synechocytis* sp. PCC6803

**DOI:** 10.1007/s11120-024-01112-7

**Published:** 2024-07-22

**Authors:** Anne-Christin Pohland, Gábor Bernát, Stefan Geimer, Dirk Schneider

**Affiliations:** 1 Department of Chemistry, Biochemistry, Johannes Gutenberg University Mainz, Hanns-Dieter-Hüsch-Weg 17, Mainz, 55128 Germany; 2 Institute of Molecular Physiology, Johannes Gutenberg University Mainz, Mainz, Germany; 3 HUN-REN Balaton Limnological Research Institute, Tihany, Hungary; 4 Cell Biology and Electron Microscopy, University of Bayreuth, Bayreuth, Germany

**Keywords:** *Synechocystis*, Mg^2+^ limitation, PQ pool, Electron transport, ΔpH, Acridine orange

## Abstract

Mg^2+^, the most abundant divalent cation in living cells, plays a pivotal role in numerous enzymatic reactions and is of particular importance for organisms performing oxygenic photosynthesis. Its significance extends beyond serving as the central ion of the chlorophyll molecule, as it also acts as a counterion during the light reaction to balance the proton gradient across the thylakoid membranes. In this study, we investigated the effects of Mg^2+^ limitation on the physiology of the well-known model microorganism *Synechocystis* sp. PCC6803. Our findings reveal that Mg^2+^ deficiency triggers both morphological and functional changes. As seen in other oxygenic photosynthetic organisms, Mg^2+^ deficiency led to a decrease in cellular chlorophyll concentration. Moreover, the PSI-to-PSII ratio decreased, impacting the photosynthetic efficiency of the cell. In line with this, Mg^2+^ deficiency led to a change in the proton gradient built up across the thylakoid membrane upon illumination.

**Supplementary Information:**

The online version contains supplementary material available at 10.1007/s11120-024-01112-7.

## Introduction

Mg^2+^ is the most abundant divalent cation in living cells (Wacker 1969). In mammalian cells, the total Mg^2+^ concentration ranges between 17 and 20 mM (Romani and Scarpa 2000), whereas it is reportedly higher in bacterial cells where it ranges between 15 and 200 mM (Lusk et al. 1968; Silver 1969; Kung et al. 1976; Moncany and Kellenberger 1981). Within cells, most of the Mg^2+^ is bound to macromolecules (Flatman 1984), and only ~ 4 mM Mg^2+^ is “free” (Lusk et al. 1968). It is estimated that around 50% of the cellular Mg^2+^ is bound to ATP (Maguire and Cowan 2002), forming an ATP-Mg^2+^ complex, the biologically active ATP form. This makes Mg^2+^ extremely important for cellular bioenergetics. In plants, Mg^2+^ is an essential macronutrient (Merhaut 2007) and of particular importance for photosynthesis, as Mg^2+^ is part of chlorophyll (Chl) (Willstätter 1906), an essential pigment involved in light harvesting and photosynthetic light reactions. Consequently, reducing the amount of available Mg^2+^ results in a reduced amount of Chl in both algae and plants (Finkle and Appleman 1953; Kobayashi and Tanoi 2015; Peng et al. 2019; Giraldo et al. 2021). In line with a reduced Chl content, a decrease in the maximum quantum yield of photosystem II (PSII) has been observed in some plants (Hermans et al. 2004; Yang et al. 2012; Tang et al. 2012; Tränkner and Jamali Jaghdani 2019), as well as reduced photosynthetic CO_2_ fixation rates (Cakmak and Kirkby 2008). Additionally, the overall PSI activity was decreased due to a significantly reduced PSI abundance (Hermans et al. 2004; Farhat et al. 2015).

Plastids have evolved from a cyanobacterial ancestor (Schimper 1883; Mereschkowsky 1905; Sagan 1967; Gray 1989), and consequentially, the structures of modern-days cyanobacteria and chloroplasts are highly similar in many aspects. Cyanobacteria have a unique and highly differentiated internal membrane system compared to other bacteria. The cyanobacterial cell envelope has a typical Gram-negative membrane organization, consisting of an inner cytoplasmic membrane (CM), which is equivalent to the chloroplast inner envelope membrane, a peptidoglycan layer, and an outer membrane (OM). Furthermore, most cyanobacteria contain a separated internal membrane system that is also found in chloroplasts, the thylakoid membranes (TMs), where the pigment-protein complexes of the photosynthetic electron transport network are located (Liu 2016). As in plants, Mg^2+^ is an essential nutrient also in cyanobacteria (Pohland and Schneider 2019), and the lower limit for growth of the cyanobacterium *Synechococcus elongatus* (*S. elongatus*, formerly: *Anacystis nidulans*) is around 5 µM Mg^2+^ (Utkilen 1982). Mg^2+^ deficiency resulted in a lowered amount of total proteins in the cyanobacteria *Cyanothece* strain 16Som2 and *Cyanospira capsulata*, accompanied by a significant enhancement of the exopolysaccharide (EPS) production in the latter (De Philippis et al. 1991). Furthermore, as observed in plants, the PSII activity was also reduced in the cyanobacterium *Arthrospira platensis* Gomont 1892 when cells were grown in medium with a low Mg^2+^ content (Urek and Kerimoglu 2019).

TMs are composed of four major lipids, two of which have a negatively charged head group (Sakurai et al. 2006). This results in a negatively charged membrane surface, which is mainly screened by loosely bound Mg^2+^ ions (Pottosin and Dobrovinskaya 2015; Kaňa and Govindjee 2016). In plants, Mg^2+^ is crucial for grana stacking, and the observed altered photosynthesis at low Mg^2+^ concentrations might be caused by the disruption of grana stacks (Hall et al. 1972; Jennings et al. 1978).

During the TM-associated photosynthetic light reaction, light energy is captured and used to drive electron transport from water to nicotinamide adenine dinucleotide phosphate through photosynthetic pigment-protein complexes, i.e., PSII, the cytochrome *b*_6_*f* complex, and PSI, as well as mobile electron carriers. The photosynthetic electron transport is tightly coupled to H^+^ translocation from the chloroplast stroma or the cyanobacterial cytoplasm, respectively, into the thylakoid lumen, resulting in acidification of the lumen and generation of a proton gradient (ΔpH) across the membrane, as initially proposed by Peter Mitchell (Mitchell 1961). A Mg^2+^ efflux from the chloroplast TM lumen electrically compensates for the light-generated ΔpH formation in plant chloroplasts (Dilley and Vernon 1965; Hind et al. 1974; Barber et al. 1974; Chow et al. 1976; Portis and Heldt 1976). Besides counterbalancing the ΔpH across TMs, Mg^2+^ can also regulate the activity of several chloroplast enzymes involved in CO_2_ fixation, such as Ribulose-1,5-bisphosphate carboxylase/oxygenase, fructose 1,6-bisphosphatase, and sedoheptulose 1,7-bisphosphatase (Portis et al. 1977; Portis 1992). Although not yet experimentally demonstrated, the intracellular Mg^2+^ concentration may also vary in cyanobacteria depending on the light conditions, as observed in chloroplasts. Moreover, unlike in chloroplasts, there is active respiratory electron flow in cyanobacteria, whose components are also localized in the TMs (for a review, see (Mullineaux 2014). This results in a ΔpH of about 2 units across the TM already in the dark (Peschek et al. 1985; Belkin et al. 1987), albeit the thylakoid lumen is further acidified upon illumination, with a decrease of at least 0.5 pH units (Belkin et al. 1987; Teuber et al. 2001).

In contrast to plants, the research on cyanobacteria has thus far primarily focused on the impact of limited Mg^2+^ availability on growth and biomass production (Utkilen 1982; De Philippis et al. 1991; Philippis et al. 1993), while its effects on photosynthetic light reactions have not yet been explored in detail.

Here, we have analyzed the impact of Mg^2+^ limitation on growth and photosynthetic performance of the cyanobacterium *Synechocystis* sp. PCC6803 (from hereon: *Synechocystis*). When *Synechocystis* wild type cells were grown under Mg^2+^ limitation, both cell growth and photosynthetic activity were affected, in line with a reduced Chl *a* content. Furthermore, measuring light-induced pH changes using the fluorescent dye acridine orange (AO) revealed a distinct change in the pH gradient across the TM under Mg^2+^ limiting conditions. This highlights the involvement of Mg^2+^ in counterbalancing the ΔpH established across the TMs.

## Materials and methods

### Growth conditions

For the growth analyses, 25 mL cultures of glucose-tolerant *Synechocystis* wild type cells were grown in 50 mL Erlenmeyer flasks in a temperature-controlled incubation shaker (Multitron HT, Infors (Bottmingen, Switzerland) at 130 rpm and 30 °C under constant illumination of 120 µmol photons m^− 2^ s^− 1^. *Synechocystis* cells were grown without glucose either in a modified BG11 medium (modified from (Rippka et al. 1979) see Supplementary Table 1) including 300 µM Mg^2+^ and 5 mM HEPES/KOH pH 8.0 (from now on BG11) or, for growth under Mg^2+^ limiting conditions, in BG11 with decreased Mg^2+^ concentration where MgSO_4_ was replaced by Na_2_SO_4_ and a defined amount of Mg^2+^ (MgCl_2_) was added afterwards. Here, the final Mg^2+^ concentration ranged from 10 to 70 µM. Growth of *Synechocystis* was monitored by measuring the optical density at 750 nm (OD_750_). For each growth condition, three biological replicates were monitored.

For all other analyses, cyanobacterial cells were grown photoautotrophically at constant cell density (OD_720_ = 1.0) in a multi-cultivator (Multi-Cultivator MC 1000-OD, Photon Systems Instruments (PSI), Drásov, Czechia) equipped with a Turbidostat TS-1000 (PSI, Drásov, Czechia), bubbled with 5% (v/v) CO_2_ in air at 30 °C (controlled by a Cooling Unit AC-710 (PSI, Drásov, Czechia)). The intensity of the (white) growth light was set to 30 µmol photons m^− 2^ s^− 1^. Cells were grown either in BG11 with 300 µM Mg^2+^ or, under Mg^2+^ limitation, in BG11 media supplemented with 50 µM Mg^2+^. Prior to the measurements, cells were centrifuged at 1800 *g* at room temperature (RT) for 10 min, and the cell pellet was solubilized in fresh medium with the OD_750_ adjusted to 2.0.

### Electron microscopy

Electron microscopic (EM) images were acquired by using whole cells grown under high and low Mg^2+^ conditions. 20 mL of a cell suspension was spun down at 1800 *g* for 10 min at RT. The supernatant was removed, and the cells were washed with 20 mM HEPES/KOH pH 7.0. After another centrifugation, the cells were resuspended in the same medium with a final OD_750_ of 5.0. The suspension was then mixed 1:1 with 10% glutaraldehyde. The fixed samples were sedimented at 3300 *g* for 10 min, washed with bi-distilled (bidest.) water at RT for 10 min, and then spun down again. The cells were post-fixed with 2% OsO_4_ (w/v in bidest. water) for 120 min at 4 °C. Thereafter, the samples were centrifuged for 10 min at 6600 g and washed with bidest. water three times. Cells grown at low Mg^2+^ conditions were infiltrated with 20% bovine serum albumin for 120 min at 4 °C, centrifuged for 10 min, and fixed in 5% glutaraldehyde in 0.05 M phosphate buffer pH 7.5 to obtain a dense pellet. All samples were then taken up in 2% agar, cut into small blocks, and washed in bidest. water for 5 min. The samples were dehydrated in ethanol of increasing concentrations. After dehydration, the solvent was replaced with a gradually increasing concentration of epon resin. After that, the samples were left in an embedding capsule for 2–3 days at 60 °C. Ultrathin sections were cut with an Ultracut EM UCT ultramicrotome (Leica Microsystems, Wetzlar, Germany) using a diamond knife (Type Ultra 45°, Diatome, Biel, Switzerland). Sections were collected on pioloform-coated copper slotted grids (Plano, Wetzlar, Germany) and stained with uranyl acetate and lead citrate (Reynolds 1963). Cells were imaged using a JEM-1400Plus transmission electron microscope (JEOL (Tokyo, Japan) operated at 80 kV and equipped with a JEOL Ruby CCD camera (3,296 × 2,472 pixels).

### Cell counting

For cell counting, cultures were diluted 20-fold in the appropriate growth medium, and 10 µL were loaded into a counting chamber (Thoma scale). After cells had settled, cells were counted regularly to determine the mean cell density of at least three biological replicates.

### Determination of pigment composition

To determine the Chl *a* and carotenoid (Car) contents, an equivalent of 500 µL culture with an OD_750_ of 2.0 was harvested *via* centrifugation for 10 min at 16,000 *g* at RT. The supernatant was discarded, and 1.0 mL of 100% methanol was added to the pellet. Then, the sample was rigorously vortexed and incubated on a platform shaker (Duomax 1030 Heidolph, Schwabach, Germany) at RT for 15 min. After an additional centrifugation step for 10 min at 16,000 *g* and 4 °C, the absorption (A) of the supernatant at 665.2 nm and 652 nm (for Chl *a*), and 470 nm and 720 nm (for Car) was measured in a standard 10 mm polystyrene cuvette using a Perkin Elmer, Lambda 435 spectrophotometer (PerkinElmer, Rodgau, Germany). The Chl *a* concentration was determined according to (Porra et al. 1989):

1 $$\:Chl\:a\:\left[\frac{\mu\:g}{mL}\right]\:=\:16.26*{A}_{665.2}-8.54*{A}_{652}\:$$

The Car concentration was determined according to (Zavrel et al. 2015):

2 $$\:Car\left[\frac{\mu\:g}{mL}\right]\:=\:\left[\frac{\text{1,000}\:({A}_{470}\:-\:{A}_{720})\:-\:2.86\:\left(Chl\:a\:\left[\frac{\mu\:g}{mL}\right]\right)}{221}\right]\:$$

### Whole-cell absorbance spectra

Spectra of whole cells were recorded using a Perkin-Elmer Lambda 35 (PerkinElmer, Rodgau, Germany) spectrophotometer equipped with an integrating sphere. Spectra were recorded in the 300–800 nm spectral region and the absorbance value at 800 nm was subtracted. After normalization to the Chl *a* absorbance at 678 nm the molar phycocyanin (PC)-to-Chl *a* ratio was calculated according to (Rakhimberdieva et al. 2001).

3 $$\:PC\::\:Chl\:a\:=\frac{4.9\:{A}_{625}\:-\:2.1\:{A}_{652}\:-\:0.8\:{A}_{678}}{0.1\:{A}_{625}\:-\:0.7\:{A}_{652}+15.8\:{A}_{678}}\:\:$$

### Low temperature (77 K) fluorescence emission spectroscopy

Low-temperature fluorescence emission spectra were recorded using an Aminco Bowman Series 2 fluorimeter equipped with a 77 K accessory. 1 mL of a cell suspension with an OD_750_ of 2.0 was filled into a glass tube and shock frozen in liquid nitrogen. Chl *a* and phycobilisomes (PBS) were excited at 435 nm and 580 nm, respectively. Spectra were recorded in the 630–760 nm spectral range. The spectra were baseline corrected and normalized to the 695 nm and 665 nm emission upon Chl *a* and PBS excitation, respectively. For further evaluation, the peak areas of the PSII and PSI emissions, centered at 695 nm and 725 nm, respectively, upon Chl *a* excitation were integrated using the Fityk curve fitting software (Wojdyr 2010). The ratio of these areas can be considered equivalent to the PSII : PSI molar ratio (Murakami 1997). The cellular PSI and PSII contents (in mol cell^− 1^), in turn, were estimated as described in (Luimstra et al. 2019), which approach exploits this ratio, as well as the cellular Chl *a* content (in mol cell^− 1^) and number of Chl *a* molecules per PSI (100) and PSII (35) reaction center (Jordan et al. 2001; Umena et al. 2011; Malavath et al. 2018; Gisriel et al. 2022).

4 $$PS{I_{Cell}} = \>{{\left[ {Chl\>a} \right]\>} \over {100 + 35\>/\>\left( {PSI\>:\>PSII} \right)}}$$

5 $$PSI{I_{Cell}} = \>{{\left[ {Chl\>a} \right]\>} \over {100\>\left( {PSI\>:\>PSII} \right) + 35}}$$

### Oxygen (O_2_) yield measurements

Photosynthetic O_2_ evolution was determined in a custom-made, thermo-regulated chamber using a fiber-optic O_2_ meter (PreSens, Regensburg, Germany) at 30 °C. The optode was calibrated with air-saturated (100% O_2_) and O_2_-free water (0% O_2_, obtained upon the addition of sodium thiosulfate). Samples were dark incubated for 15 min prior to the measurements. 10 mM sodium bicarbonate (NaHCO_3_) was added as an electron acceptor. The recordings started with measuring the O_2_ consumption in the dark, followed by monitoring O_2_ production upon strong light exposure (3000 K; KL 2500 LCD, Schott, Mainz, Germany). The net O_2_ evolution was determined by subtracting the slope of the signal during the dark from the slope of the subsequent O_2_ evolution.

#### Pulse amplitude modulation (PAM) fluorometry

**Fig. 1:**
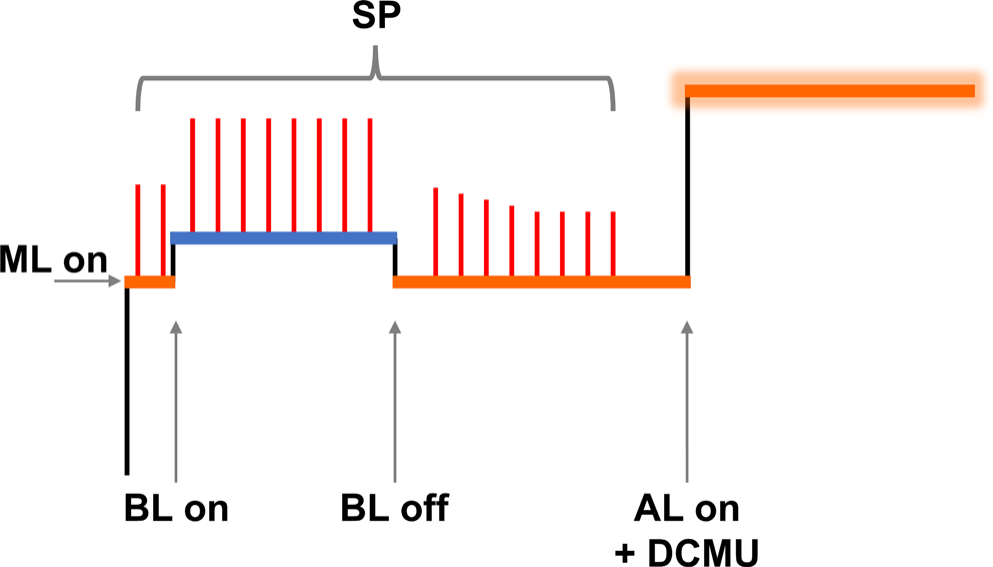
Schematic view of a fluorescence induction curve with the applied lights and saturating pulses on top. ML, Measuring light (I = 2 µmol photons m^–2^ s^–1^); BL, weak blue light (I = 29 µmol photons m^–2^ s^–1^); SP, saturating pulse (I = 10,000 µmol photons m^–2^ s^–1^); AL, strong red actinic light (I = 187 µmol photons m^–2^ s^–1^); DCMU, 3-(3,4-dichlorophenyl)-1,1-dimethylurea

PSI and PSII associated quantum yields were determined using a Dual-PAM-100 measuring system equipped with Dual-E and DUAL-DR modules (Walz GmbH, Effeltrich, Germany). The maximum quantum yield of PSII (F_v_/F_m_) (Kitajima and Butler 1975) was determined by recording fluorescence induction curves at RT (Table 1; Fig. 1) after 5 min of dark acclimation. The initial fluorescence, F_o_, and the maximal fluorescence under actinic light upon addition of 3-(3,4-dichlorophenyl)-1,1-dimethylurea (DCMU) (Campbell et al. 1998), F_m_, were used for the determination of F_v_/F_m_.

6 $$Fv/Fm\> = \>(Fm\> - \>Fo)/Fm$$

Furthermore, weak blue light was used to induce state 1 transition (Schreiber et al. 1995). During each phase 600 ms saturating light pulses were applied on top to follow the corresponding maximal signal intensity (I = 10,000 µmol photons m^–2^ s^–1^).

The effective quantum yield of PSI (Y(I), the PSI quantum yield with donor and acceptor side limitations (Y(ND) and Y(NA), respectively), and the effective PSII quantum yield (Y(II) were determined *via* recording rapid light curves by the Dual-PAM-100 system. Here, the actinic light intensities increased stepwise from 0 to 827 µmol photons m^− 2^ s^− 1^. Steady state (F_s_) and maximal (F_m_’) fluorescence levels were determined after 30 s illumination period at each light intensity where 300 ms saturating pulses with an intensity of 10,000 µmol photons m^− 2^ s^− 1^ were applied. The corresponding PSI parameters, i.e. the maximum amplitude of the P_700_ signal after far red illumination (P_m_), the steady state (P) and maximum (P_m_’) P_700_ signal under illumination were determined concomitantly. Parameters were calculated according to (Genty et al. 1989; Klughammer and Schreiber 2008).

7 $$Y\left( I \right)\> = \>(Pm' - P)/Pm$$

8 $$Y\left( {ND} \right)\> = \>\left( P \right)/Pm'$$

9 $$Y\left( {NA} \right)\> = \>(Pm - Pm')/Pm$$

10 $$Y\left( {II} \right)\> = \>(Fm' - Fs)/Fm'$$

### P_700_ re-reduction kinetics

P_700_ re-reduction kinetics were measured using a Dual-PAM-100 measuring system (Klughammer and Schreiber 1994). Complete P_700_ oxidation was achieved by a 100-ms saturating pulse (I = 10,000 µmol photons m^–2^ s^–1^). P_700_^+^ decay kinetics were fitted with single exponential functions to determine the corresponding rate constant (*k*).

### Acridine orange (AO) fluorescence measurements

The magnitude and kinetics of pH changes within *Synechocystis* cells upon illumination were monitored using the fluorescent dye AO (Teuber et al. 2001) with a Dual-PAM-100 measuring system equipped with an Acridine Orange/Yellow fluorescence emitter-detector module. For each measurement, 1.5 mL cell suspension was mixed with 100 µL of Tricine buffer (1 M, pH 8.0) and 1 µL of an AO solution (10 mM), in a 10 mm quartz cuvette. Each measurement is preceded by a 15-minute dark incubation. After starting the measurements, cells were kept for another three minutes in the dark, then red AL with an intensity of 216 µmol photons m^–2^ s^–1^ was provided for five minutes. Eventually, AO fluorescence was recorded for another two minutes in the dark. Spectra were baseline corrected and normalized to the fluorescence intensity monitored during the first three minutes in the dark.

## Results

### Mg^2+^ is essential for the cell growth of Synechocystis

To first elucidate the importance of Mg^2+^ for cell viability, *Synechocystis* cells were grown in standard growth medium as well as in media with reduced Mg^2+^ contents, and cell growth was monitored.

**Fig. 2:**
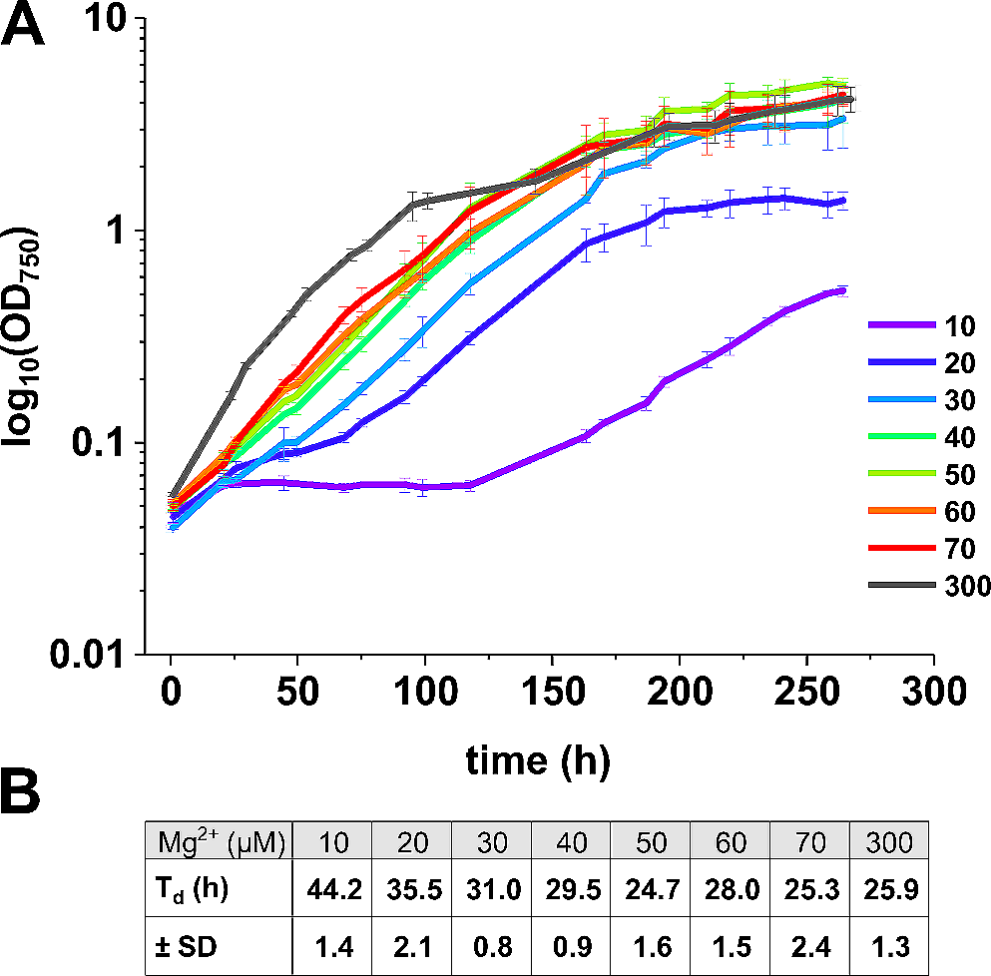
Growth of *Synechocystis* at different Mg^2+^ concentrations. (**A**) Growth curves of *Synechocystis* at different Mg^2+^ concentrations with the Mg^2+^ concentration in the growth medium (in µM) shown next to the color bars. (**B**) Doubling times (T_d_) at different Mg^2+^ concentrations. Error bars represent the standard deviation (SD) of the mean (*N* = 3)

**Fig. 3:**
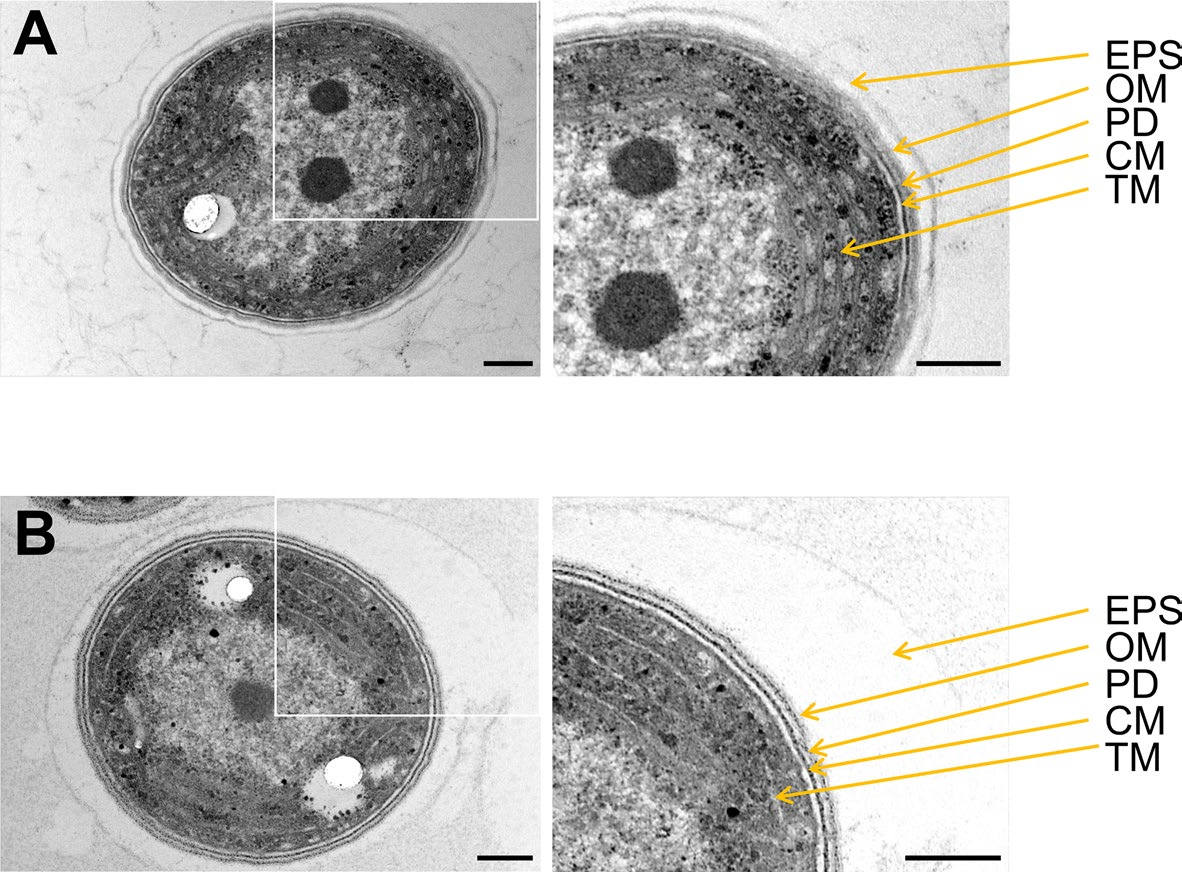
EM images of *Synechocystis* cells grown in HM or LM medium. Compared to standard growth conditions (**A**), whole cell images show an extensive exopolysaccharide layer (EPS) when cells were grown under Mg^2+^-limiting conditions (**B**). Images on the right show magnification of the boxed areas of the corresponding images on the left. EPS, exopolysaccharide; OM, outer membrane; PD, peptidoglycan layer; CM, cytoplasmic membrane; TM, thylakoid membrane. Scale bars = 200 nm

BG11, the standard growth medium of *Synechocystis*, contains a Mg^2+^ concentration of about 300 µM (Rippka et al. 1979); we refer to this as “high Mg^2+^” (HM) from hereon. As shown in Fig. 2A, *Synechocystis* has the highest growth rates at this condition. With decreasing Mg^2+^ concentrations *Synechocystis* is still able to grow, even at a Mg^2+^ concentration of as low as 10 µM, albeit at low Mg^2+^ concentrations the maximal culture densities, expressed as OD_750_, decreased significantly and cells reached a stationary phase later compared to standard conditions. When grown in the presence of 10 µM Mg^2+^ in the growth medium the doubling time (T_d_) was about 1.7 times longer (44.2 ± 1.4 h) compared to the control (HM, T_d_ = 25.9 ± 1.3 h) (Fig. 2B). Furthermore, a prolonged initial lag phase can be observed at Mg^2+^ concentrations below 40 µM. Above this concentration, the growth characteristics were similar to HM conditions, and thus, we decided to use 50 µM Mg^2+^ (low Mg^2+^, LM) in the culture medium for all subsequent measurements to ensure the survival of *Synechocystis* cells under Mg^2+^ limiting growth conditions. Noteworthy, while *Synechocystis* cultures grew well under LM conditions, a massive EPS-layer, composed of secreted (exo) polysaccharides (EPS), surrounding the individual cells accumulated when cells were grown under LM conditions (Fig. 3).

### Content and composition of pigment-containing protein complexes involved in photosynthesis

**Fig. 4:**
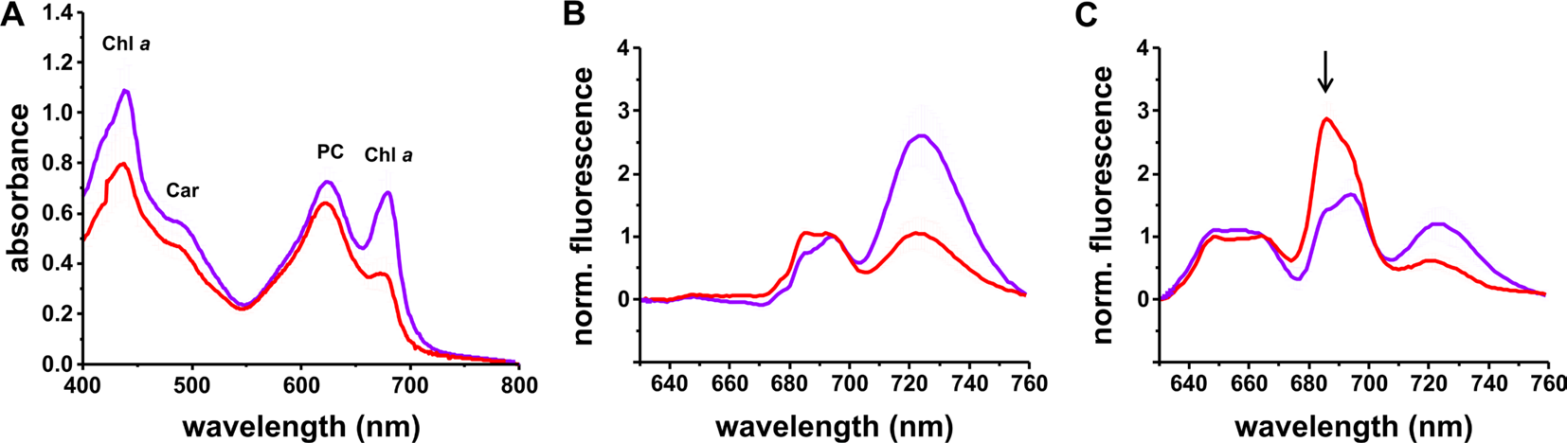
Absorbance and 77 K fluorescence spectra of intact *Synechocystis* cells grown under HM or LM conditions. (**A**) In the absorbance spectra, the reduction in Chl *a* concentration under LM (red) compared to HL (blue) conditions is visible as decreased peak intensities at 437 nm and 678 nm. An increase in the PC-to-Chl *a* ratio can also be observed. The absorbance maxima of the respective pigments are indicated for comparison. Chl *a*, chlorophyll *a*; Car carotenoids; PC, phycocyanin. Error bars represent means ± SD (*N* = 6 (HM); *N* = 3 ( LM)). (**B**) 77 K fluorescence emission spectra were recorded upon Chl excitation at 435 nm and normalized to 695 nm. Mg^2+^ limitation (red) resulted in a decreased PSI-to-PSII ratio. (**C**) 77 K spectra after PBS excitation at 580 nm and normalization to 665 nm revealed altered PC-to-PSI and PC-to-PSII ratios under Mg^2+^ limitation (red). The arrow marks the peak that presumably shows uncoupled PBS (as further discussed in the text). Error bars represent means ± SD (*N* = 5 (HM); *N* = 3 (LM))

When *Synechocystis* cells were grown in LM medium, the absorbance spectra of whole cells differed significantly from spectra of HM-grown cells: the characteristic Chl *a* absorbance maxima at 437 nm and 678 nm were severely reduced, indicating a highly reduced Chl *a* content, which suggests a lower cellular abundance of Chl *a*-containing protein complexes, i.e. PSs (Fig. 4A). The PC-to-Chl *a* ratio increased from 0.19 ± 0.03 under HM conditions to 0.38 ± 0.05 under LM conditions calculated from normalized spectra, as described in Material and Methods.

The decreased Chl *a* content observed in the absorbance spectra of LM cultures was confirmed by spectroscopic analyses of methanolic cell extracts (Supplementary Fig. 1). The cellular Car content, which is visible as a shoulder at 480 nm in the whole cell absorbance spectra (Fig. 4A), was also reduced in the LM cultures, albeit to much lower extents (Supplementary Fig. 1B). Thus, the calculated Chl *a*-to-Car ratio was lower in LM grown cells compared to HM grown (Supplementary Fig. 1C).

Next, we analyzed the relative abundance of the two PSs, as well as energy transfer from (PBS) to PSI and PSII *via* low temperature (77 K) fluorescence spectroscopy. Cyanobacteria regulate the energy distribution between the two PSs by adjusting their soluble antenna systems, the PBS which consist of six rods of phycocyanin (PC) and one core of allophycocyanin (APC) (Kirilovsky et al. 2014; Calzadilla and Kirilovsky 2020). PBSs are primarily associated with PSII when the PQ pool is oxidized (state I) and with PSI when the PQ pool is reduced (state II) (Mullineaux and Allen 1990).

Upon excitation of Chl *a* at 435 nm, the HM cultures exhibit fluorescence emission spectra typical for *Synechocystis* (Fig. 4B) with main emission peaks at around 685 nm, 695 nm, and 725 nm, which originate from the PSII core antennas CP43 and CP47 and (to some extent) the PBS terminal emitter (at 685 nm), CP47 of PSII (695 nm), and PSI (725 nm), respectively (Rijgersberg and Amesz 1980; Shen et al. 1993; Andrizhiyevskaya et al. 2005). Under LM conditions, the decrease in the relative fluorescence emission at 725 nm indicates a reduced relative amount of PSI. This is in good agreement with the observation that about 90% of the total Chl *a* in *Synechocystis* is bound by PSI (Jordan et al. 2001; Umena et al. 2011; Zakar et al. 2018; Malavath et al. 2018). To quantify the relative changes in the cellular abundance of the PSs, we next calculated the photosystem per cell content as described in Material and Methods. According to the results, the number of PSII/cell hardly changed under LM conditions compared to HM conditions (0.030 ± 0.002 10^− 18^ mol/cell (*N* = 4) vs. 0.031 ± 0.002 10^− 18^ mol/cell (*N* = 3), respectively). In contrast, the number of PSI decreased significantly from (0.27 ± 0.03) × 10^− 18^ mol/cell (HM, *N* = 4) to (0.10 ± 0.02) × 10^− 18^ mol/cell (LM, *N* = 3). In line with the lowered cellular PSI content, a decreased energy transfer from PBS to PSI was observed upon PBS excitation at 580 nm (Fig. 4C). Meanwhile, the fluorescence emission at 685 nm and 695 nm increased, indicating an increased PBS-to-PSII energy transfer. Nevertheless, as the PBS terminal emitter may also contribute to the 685 nm fluorescence emission signal, this increased fluorescence emission intensity rather suggests an increased abundance of uncoupled PBSs, as observed previously (Mullineaux 1994; Barthel et al. 2013; Kłodawska et al. 2020), in line with an increased relative PC content (Fig. 4A).

### The activity of photosystem I and II

While an altered PSI-to-PSII ratio was indicated, it remained unclear whether Mg^2+^ limitation has an impact of the activity of the respective PS.

The activity of PSII was analyzed by recording fluorescence induction curves. While the method, in principle, probes the redox state of Q_A_, the primary electron acceptor of PSII, it also probes the redox state of the PQ pool as well as the status of the entire electron transport chain and the antenna systems (Schreiber 2004).

**Fig. 5:**
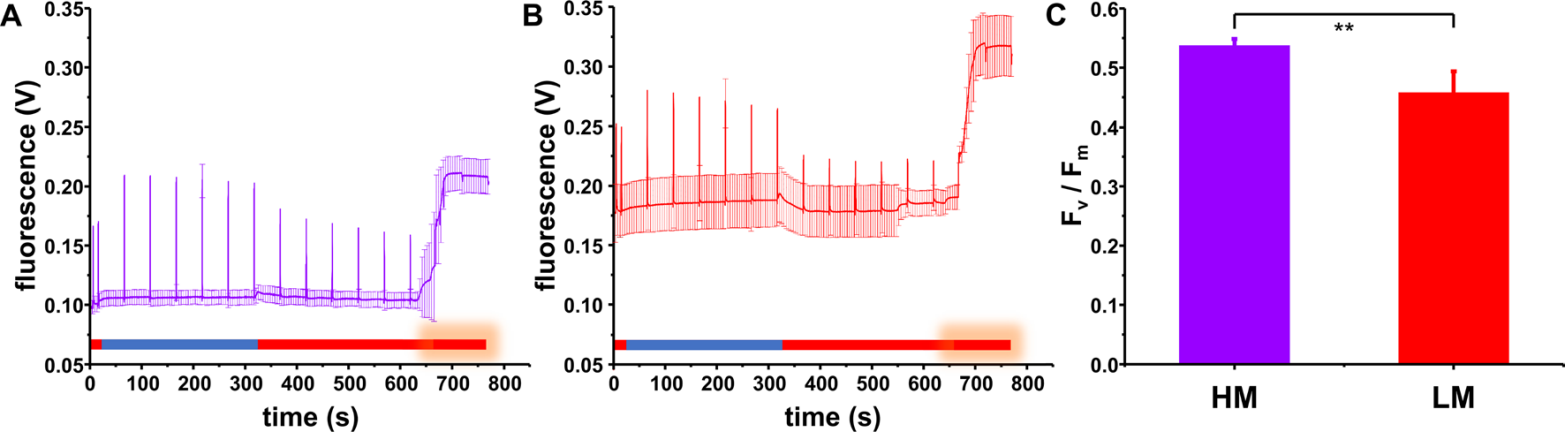
Fluorescence induction curves and the maximal PSII quantum yield of *Synechocystis* cells grown under HM and LM conditions. (**A**, **B**) Averaged traces of fluorescence induction curves measured with HM (**A**) or LM (**B**) cultures. The color bar below the traces indicates the light quality as described in Material and Methods. For clarity, only every 150th data point is displayed. After dark incubation cells were illuminated with low intensity measuring light, and a saturating light pulse was given. Then weak blue light was turned on to induce state I transitions. Saturating light pulses were given repeatedly to monitor changes in maximum fluorescence. Next the blue light was turned off, and saturating light pulses were kept being given to follow the transition back to state II. After the addition of DCMU, continuous actinic light was turned on, F_m_ was determined and the maximal quantum yield was estimated (**C**). The higher PC-to-Chl *a* ratio and the abundance of uncoupled PBS likely resulted in an overall higher signal under LM conditions. Error bars represent means ± SD (*N* = 4 (HM); *N* = 6 (LM)). Significant differences (according to Student’s t-test) are indicated as: ** *p* < 0.01

At first, the maximum PSII quantum efficiency F_v_/F_m_ was determined, an often used proxy to characterize the PSII performance in plants (Maxwell and Johnson 2000) as well as in cyanobacteria (Campbell et al. 1998). As shown in Fig. 5, both the initial (“minimal”) fluorescence yield F_o_ as well as the maximum fluorescence yield upon illumination (F_m_´) were higher in LM grown *Synechocystis* cells compared to cells grown under HM conditions. Yet, as the applied measuring light (λ_ex_ = 620 nm) excites not only Chl *a* molecules but also PBSs, PBS fluorescence emission may also significantly contribute to the higher F_o_ value (Campbell et al. 1996). The observed increase in the PC-to-Chl *a* ratio seen in the absorbance spectra (Fig. 4A) and the higher amount of uncoupled PBS observed in the 77 K fluorescence emission measurements (Fig. 4C) are in line with this assumption. In the case of the HM cultures, the F_m_´ values determined under blue light illumination were very similar to the values determined under red light in the presence of DCMU, while in the LM cultures, these were substantially lower. This suggests a less effective state I transition in the LM cultures, most likely due to the lower abundance of PSI, which is crucial for the oxidation of one key element of state transitions, the PQ pool.

**Fig. 6:**
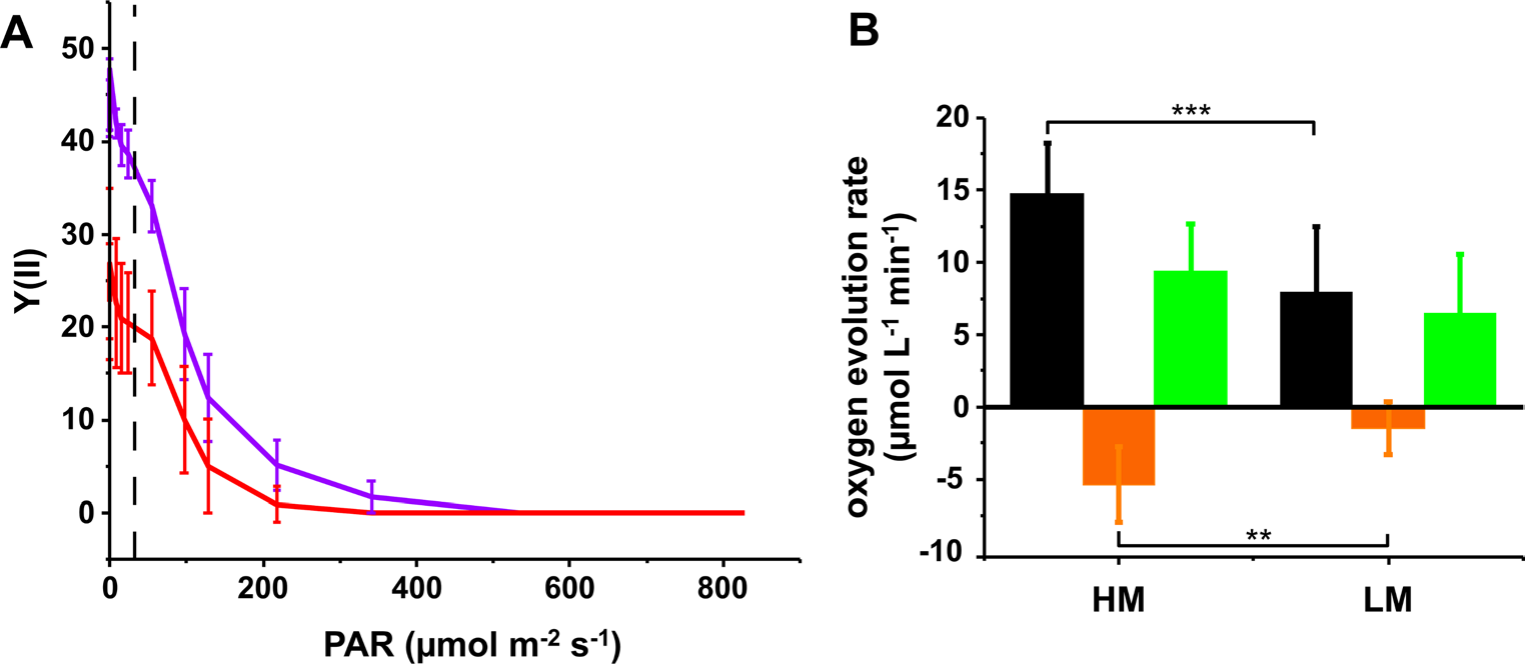
PSII activity assessed via rapid light curves and oxygen evolution measurements. (**A**) The PSII quantum yields Y(II) were significantly lower when cells were grown under LM (red) compared to HM (blue) conditions The growth light intensity is marked with a dashed line. Error bars represent means ± SD (*N* = 3 (HM); *N* = 5 (LM)). (**B**) O_2_ evolution (green), O_2_ consumption (orange), and net O_2_ production (black) rates determined analysing HM and LM grown cultures, respectively, under high light at OD_750_ = 2.0. Error bars represent means ± SD (*N* = 5 (HM); *N* = 7 (LM)). Significant differences (according to Student’s t-test) are indicated as: ** *p* < 0.01 and *** *p* < 0.001

In line with this, in LM grown cells some fluorescence quenching was observed upon blue light illumination (Fig. 5B). The F_v_/F_m_ values (maximal PSII quantum yields) calculated based on F_o_ and the maximum fluorescence yields obtained in the presence of DCMU (F_m_) were slightly smaller in the LM compared to the HM cultures (Fig. 5C). However, this is most likely due to the enhanced F_o_ level. To further analyze the PSII activity, we next applyed rapid light curves to probe energy conversion efficiency at different light intensities. The effective quantum yield of PSII (Y(II)), which indicates the quantity of absorbed light that is “used” by PSII, was decreased when cells were grown under LM conditions (Fig. 6A).

Next, we measured O_2_ evolution rates using intact *Synechocystis* cells grown either under LM or HM conditions (Fig. 6B). As the PSII contents per cell were rather similar, yet the Chl *a* concentration differed between the HM and LM cultures, we decided to express the O_2_ yields as µmol O_2_ L^− 1^ min^− 1^ at an OD_750_ = 2.0 (see Supplementary Fig. 4 for oxygen evolution rate per cell). Compared to HM conditions, the net oxygen evolution rate was significantly reduced under LM conditions, in line with the Y(II) measurements. Yet, Mg^2+^-limitation influenced respiration much more than O_2_ production, thus, the observed lower apparent O_2_ production rates in cells grown under LM conditions were mainly due to reduced O_2_ consumption, in excellent agreemen with the observation that the PSII content per cell is largely unchanged.

**Fig. 7:**
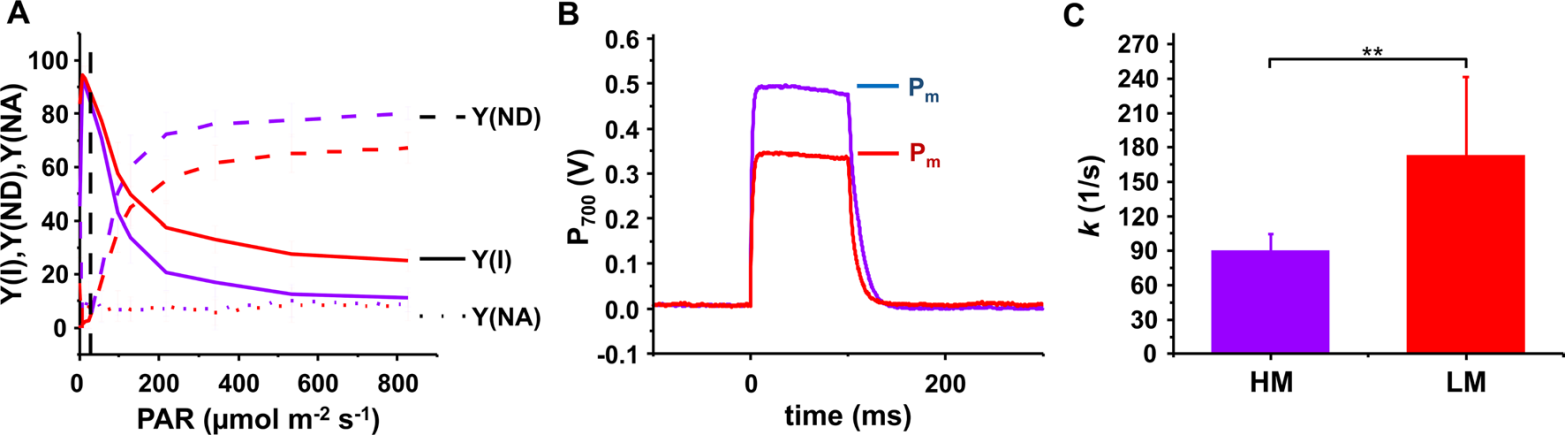
PSI activity assessed via rapid light curves and P_700_/ P_700_^+^ oxidation/re-reduction kinetics (**A**) Light intensity dependence of PSI activity: The effective quantum yield Y(I) is affected by donor side limitation Y(ND), which means a lack of electrons to reduce P_700_^+^, and by acceptor side limitation, in which P_700_ cannot be oxidized to P_700_^+^ as the acceptor is reduced. Y(I) (solid line), Y(ND) (dashed line), and Y(NA) (dotted line) in HM (purple) or LM (red) grown *Synechocystis*. The growth light intensity is marked with the dashed black vertical line. Higher Y(I) values were determined under LM conditions due to lowered Y(ND). Error bars represent means ± SD (*N* = 3 (HM); *N* = 5 (LM)). (**B**) P_700_/P_700_^+^ oxidation/re-reduction kinetic traces of HM (purple) and LM (red) grown *Synechocystis.* Oxidation of P_700_ was induced by a 100 ms saturating pulse with an intensity of 10,000 µmol photons m^− 2^ s^− 1^. 10 and 5 independent traces were baseline corrected and averaged from HM and LM grown cultures, respectively. The reduced abundance of PSI in the LM cultures is manifested as a reduced P_m_ value. (**C**) Traces from (**B**) were fitted with a single exponential decay, and the determined rate constants (*k* [1/s]) are shown. P_700_^+^ re-reduction was faster when cells were grown under Mg^2+^ limitation consistent with the lower Y(ND). Error bars represent means ± SD. Significant differences (Student’s t-test) are indicated as: ** *p* < 0.01

While the activity of PSII was only slightly affected, we subsequentely focused on the activity of PSI. Due to a significant decrease in the amount of PSI under LM conditions, we anticipated a changed activity. To study the electron transport to and from PSI at different light intensities, we applied rapid light curves. With increasing light intensities the effective quantum yield of PSI (Y(I)) decreases less prominently in the LM grown cultures compared to the HM grown cells, due to a lower donor side limitation, Y(ND), while limitations at the acceptor side Y(NA) were negligible under both conditions (Fig. 7A). This implies that in cells grown under LM conditions, electron shortage is less pronounced and more electrons arrive at PSI to reduce P_700_^+^ to P_700_. The lowered Y(ND) is in agreement with a reduced PSI content. The reduced number of oxidizable P_700_ centers in LM grown cells is also evident by two features visible in P_700_/P_700_^+^ oxidation/re-reduction kinetic measurements (Fig. 7B, C): (i) by the decreased P_m_ intensities /Fig. 7B) and (ii) by the faster P_700_ re-reduction kinetics (Fig. 7C) due to the decreased PSI-to-PSII ratio (Fig. 4B).

### Mg^2+^ limitation affects the ΔpH established across thylakoid membranes

**Fig. 8:**
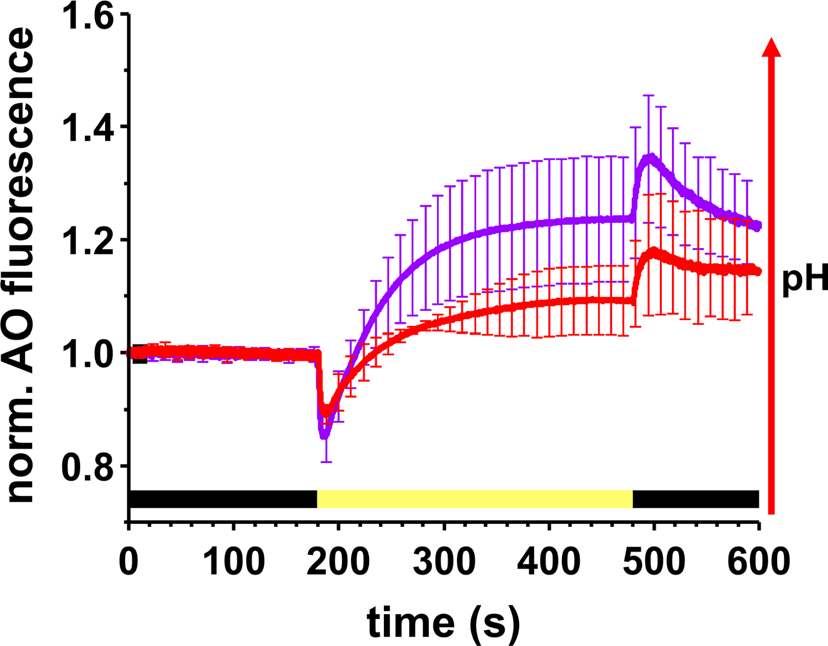
ΔpH formation across the thylakoid membranes in HM and LM grown *Synechocystis* cells. Changes in the trans-thylakoid pH gradient were monitored *via* AO fluorescence in HM (purple) or LM grown (red) *Synechocystis*. The color bar below the curve indicates the dark (black) - light (yellow) - dark (black) transition. After recording AO fluorescence in the dark for three minutes, cells were exposed to actinic light for five minutes, followed by a two-minute dark period. Error bars represent means ± SD (*N* = 15 (HM); *N* = 4 (LM))

Mg^2+^ depletion affects the PS stoichiometry, and thus electron transport properties, plus Mg^2+^ is essential to counterbalance the ΔpH built-up across the TM driven by photosynthetic electron transport (Dilley and Vernon 1965; Hind et al. 1974; Barber et al. 1974; Chow et al. 1976; Portis and Heldt 1976). Consequentely, we next analyzed whether Mg^2+^ limitation affected formation of the ΔpH using the fluorescent dye acridine orange (AO) (Fig. 8), as described in Teuber et al. (Teuber et al. 2001). Upon turning on actinic light, a rapid drop in the AO fluorescence signal intensity was observed, due to acidification of the thylakoid lumen *via* water splitting at PSII (Teuber et al. 2001), followed by a gradual fluorescence increase for two minutes until a plateau was reached.This rise in the fluorescence intensity can be interpreted as an alkalization of the cytoplasm (Teuber et al. 2001). Upon switching the light off, the fluorescence intensity rapidly increased, indicating re-alkalization of the thylakoid lumen, followed by a slow signal decrease. It should be noted that, because the periplasm was buffered to pH 8.0, the influx of protons from the periplasm into the cytoplasm was limited. Thus, a complete signal recovery cannot be expected. The overall pattern of the AO fluorescence emission changes was similar for HM and LM grown *Synechocystis* cells. However, while acidification of the TM lumen *via* water splitting was only slightly affected, in perfect agreement with the essentially unchanged PSII content per cell, a much lower plateau was reached upon illumination of LM grown cells, suggesting a reduced alkalization of the cytoplasm. Thus, a reduced amount of available Mg^2+^ apparently significantly affects generation of the ΔpH across the TM, albeit PSII acidified the thylakoid lumen to a similar extent at both HM and LM conditions.

## Discussion

Mg^2+^ is crucial for growth and development of *Synechocystis* cells. Decreasing Mg^2+^ concentrations in the growth medium led to an increased doubling time (Fig. 2), yet *Synechocystis* cells can still grow at Mg^2+^ concentrations as low as 10 µM. Such ability to adapt to very low Mg^2+^ concentrations was previously noticed for the cyanobacterium *S. elongatus* (Utkilen 1982). While growth was only little affected at Mg^2+^ concentrations of 50 µM and above, cells grown at lower concentrations did not reach saturation levels observed with cells grown at higher Mg^2+^ concentrations. The extended lag phase observed at low Mg^2+^ concentrations suggests that the cellular metabolism adapted to the new growth condition (Bertrand 2019). Since the growth of *Synechocystis* appeared to be only slightly affected in a medium containing 50 µM Mg^2+^, this concentration was used in all further measurements.

In several non-photosynthetic bacteria (Webb 1951; Brock 1962) and in the cyanobacterium *S. elongatus* (Utkilen 1982), Mg^2+^ limitation impairs cell division. While the Mg^2+^ concentration was high enough in our experiments to not affect cell division, EM images clearly revealed an enlarged EPS layer (Fig. 3). The formation of EPSs in biofilms typically correlates with the ability to cope with changing environmental conditions (Pereira et al. 2009; De Philippis et al. 2011; Rossi and De Philippis 2015). Former observations suggest a link between Mg^2+^ deficiency and increased EPS formation, as this response was also observed in the cyanobacterium *Cyanospira capsulata* (De Philippis et al. 1991) as well as in *Pseudomonas aeruginosa* (Mulcahy and Lewenza 2011). EPS chelates bivalent cations, (De Philippis et al. 2011) and it has been suggested that *Microcystis flos-aquae* C3-40 accumulates Fe^2+^/Fe^3+^ and Mn^2+^ in the polysaccharide capsule (Parker et al. 1996). Thus, the increased EPS layer may assist *Synechocystis* in accumulating Mg^2+^ in its immediate environment at LM conditions.

### Mg^2+^limitation causes changes in cell pigmentation, a reduced amount of PSI, and changed energy distribution between the photosystems

As Mg^2+^ is the central ion of Chl molecules, lowering the Mg^2+^ concentration in the growth medium was expected to affect the amount of pigments per cell, as has been observed in plant and algae chloroplasts (Finkle and Appleman 1953; Volgusheva et al. 2015; Kobayashi and Tanoi 2015; Peng et al. 2019; Giraldo et al. 2021), as well as in the cyanobacterium *S. elongatus* (Utkilen 1982). Indeed, in *Synechocystis* cells grown in LM medium the Chl *a* content was significantly reduced (Fig. 4). The concentration and the intracellular transport of Mg^2+^ are tightly regulated, and as free Mg^2+^ is involved in various cellular processes, its cellular level is of special relevance (Maguire 1990). Therefore, it is likely that the overall concentration of Chl *a* decreased in order to mobilize Mg^2+^ and to not lower the total amount of free Mg^2+^ too dramatically, as has been observed in rice (Peng et al. 2019). The decreased cellular Chl *a* content was mainly due to a reduction in PSI abundance (see above). This resulted in the observed decrease in the PSI-to-PSII ratio, as seen in the 77 K fluorescence emission spectra (Fig. 4B). A reduction in the PSI level at LM conditions appears to be reasonable in *Synechocystis*, as most Chl *a* is bound to PSI due to a high PSI-to-PSII ratio (2:1 to 5:1 under standard conditions, (Shen et al. 1993; Murakami 1997), and the observation that each PSI unit contains about 100 Chl *a* molecules (Jordan et al. 2001; Malavath et al. 2018), whereas PSII binds only about 35 (Umena et al. 2011; Gisriel et al. 2022). Analogously, a reduction in the PSI content has also been proposed/observed in plant chloroplasts under Mg^2+^ limitation (Hermans et al. 2004; Farhat et al. 2015). Such a reduction in PSI was previously observed under several stress conditions. A decrease in PSI has long been known as a response when *Synechocystis* cells are shifted from low to high light conditions, presumably to reduce the production of reactive oxygen species (Fujita 1997; Hihara et al. 1998; Sonoike et al. 2001). Furthermore, the PSI abundance is reduced when electron transfer is impaired (Schneider et al. 2001) or cells are grown under iron starvation to prevent oxidative stress (Fraser et al. 2013). Altered PSI-to-PSII ratios are often observed when cells are stressed and in fact the relative enrichment of carotenoids (Fig. 4 and Supplementary Fig. 1BC) further supports cellular stress, as carotenoids play a crucial role in preventing oxidative damage (Steiger et al. 1999; Zakar et al. 2017). In cyanobacteria, carotenoid-binding proteins are heavily involved in preventing overexcitation by regulating the energy transfer from the PBS to the PSs (Kirilovsky et al. 2014). The absorption of blue-green light by the carotenoid bound to the orange carotenoid protein (OCP) induces conformational changes, allowing the protein to bind to the PBS and dissipate excess energy as heat (Wilson et al. 2008; Gwizdala et al. 2011) and the bound carotenoids of the High Light-Inducible Carotenoid-Binding Protein Complex (HLCC) also prevent oxidative damage (Daddy et al. 2015).

Besides a changed Chl *a*-to-Car ratio, we also observed an increase in the PC-to-Chl *a* ratio at LM conditions (Fig. 4A). The decreased PSI content and the increased PC-to-Chl *a* ratio have caused a shift in the energy distribution between the two PSs, as observed via 77 K fluorescence emission spectroscopy (Fig. 4BC). Due to the increased PC-to-Chl *a* ratio and the decreased PSI amount (Fig. 4AB), PBS-to-PSII energy transfer is enhanced. Furthermore, the increased fluorescence emission at 685 nm (relative to the 695 nm) observed in the 77 K fluorescence emsission spectra upon excitation of Chl *a* at 435 nm (Fig. 4C) suggests uncoupled PBS´s, as the PBS terminal emitter also emits light at 685 nm (Mullineaux 1994; Barthel et al. 2013; Kłodawska et al. 2020). PBS uncoupling is commonly caused by environmental stresses, such as heat or high irradiance (Kirilovsky et al. 2014). This process could help preventing over-excitation of the electron transport chain (Tamary et al. 2012), which likely occurs at LM due to the altered PSI-to-PSII ratio.

### Changes in PSI-to-PSII stoichiometry alter electron transport properties in LM grown Synechocystis

The increase in the PBS to PSII energy transfer as well as the presence of potentially uncoupled PBS under LM conditions perfecely explain the higher F_o_ and F_m_ signals observed in the fluorescence induction curves (Fig. 5B). For instance, high F_o_ signals were previously observed in *Synechococcus* sp. PCC7942 when the PC-to-Chl ratio was high (Campbell et al. 1996). A reduced F_v_/F_m_ value was also previously observed in plants grown under Mg^2+^ deficiency (Hermans et al. 2004; Yang et al. 2012; Tang et al. 2012; Jamali Jaghdani et al. 2021). Generally, a decreased F_v_/F_m_ ratio is a sign of cellular stress or damage associated with PSII (Maxwell and Johnson 2000; Murchie and Lawson 2013), might indicating a reduced PSII content in LM grown cells.

However, the PSII activity, quantified as O_2_ evolution rates, under LM conditions was rather unaffected, while the O_2_ consumption rates in LM grown cells were significantly decreased (Fig. 6B). In terms of quantum efficiency, the effective quantum yields of both PSs were influenced by Mg^2+^ limitation, though to different extents. We observed a substantial decrease in the PSII quantum yield, Y(II) (Fig. 6A), in accordance with the decreased F_v_/F_m_ ratios. Such decrease in Y(II) has also been reported for barley chloroplasts (Jamali Jaghdani et al. 2021) and the cyanobacterium *Arthrospira platensis* Gomont 1892 grown at low Mg^2+^ concentrations (Urek and Kerimoglu 2019). As the PSII content per cell appears to be largely unaffected by the LM growth conditions, the decreased F_v_/F_m_ ratio can be explained by an increased basic and steady state fluorescence and/or a more reduced PQ pool upon illumination. Concomitantly, we observed a simultaneous increase in the effective quantum yields of PSI Y(I) under LM conditions (Fig. 7A), mainly due to a decrease in Y(ND), which is consistent with the decrease in the PSI-to-PSII ratio. As recently shown, the P_700_^+^ reduction kinetics mostly slowed down when the amount of PSI was decreased (Moore and Vermaas 2024), and thus the altered ΔpH across TMs might play a role in increasing the P_700_^+^ re-reduction rates. In fact, using an uncoupler results in increased electron transport rates with a simultaneous decrease in ΔpH (Evron and McCarty 2000) which, in turn, results in lower Y(ND) and faster P_700_^+^ re-reduction kinetics. In accordance, we observed a lower light-induced ΔpH across the TM under LM condition (Fig. 8). Therefore, the faster P_700_^+^ re-reduction rates (Fig. 7C) and lower Y(ND) (Fig. 7A) observed under Mg^2+^ limiting conditions are likely due to both an decreased PSI-to-PSII ratio and a lower ΔpH.

### Mg^2+^ deficiency results in a decreased transmembrane ΔpH

The above described changes, including lower F_v_/F_m_ and Y(II), along with an increased Y(I), were accompanied by an altered pattern of ΔpH formation across *Synechocystis* membranes (Fig. 8), including a much lower alkalization of the cytoplasm in cells grown under LM conditions compared to HM conditions. When a pH gradient is formed across the TMs upon illumination, the concomitantly generated transmembrane electric potential is balanced by Mg^2+^ and K^+^ flux from the lumen to the cytoplasm plus Cl^−^ flux in the opposite direction (Dilley and Vernon 1965; Hind et al. 1974; Chow et al. 1976; Lyu and Lazár 2017). Therefore, when cells were grown under LM conditions, altered Mg^2+^ concentrations in the cytoplasm and/or lumen may impair proton flux across the TM. Based on AO measurements performed with a *Synechocystis* mutant where a predominantly TM-localized potassium channel (*Syn*K) was deleted, the acidification of the lumen was less pronounced upon illumination compared to the wild type (Checchetto et al. 2012). Likewise, the reduced ΔpH observed in LM cells could well be caused by the reduced Mg^2+^ concentration, and thus, reduced Mg^2+^ flux across the TM.

According to the AO measurements, the signal increase, reflecting alkalization of the cytoplasm, was only about half in size in LM grown cells compared with cells grown under HM conditions. The light-induced alkalization of the cytoplasm is achieved by proton pumping to the TM lumen by electron transport-coupled proton translocation and also by proton extrusion through the CM (Teuber et al. 2001). Proton extrusion across the CM is the result of respiration as well as ATP hydrolysis (Teuber et al. 2001) and/or proton extrusion by the PxcA (former CotA) protein driven by a still unknown mechanism (Katoh et al. 1996; Sonoda et al. 1998; Inago et al. 2020). Therefore, the impaired respiration observed in cells grown under LM conditions (Fig. 6B) may also contribute to the alterations in the AO fluorescence signal, finally affecting the PSI activity (see above).

Upon switching the light off, a fast increase in the AO fluorescence intensity was observed, which is most likely due to a rapid re-alkalization of the lumen. According to Teuber et al. (Teuber et al. 2001), the subsequent signal decrease in the dark may reflect the consumption of redox equivalents. Since many enzymes in the Calvin–Benson–Bassham cycle require Mg^2+^ as a cofactor and alkalization of the lumen for proper function (Lorimer et al. 1976; Flügge et al. 1980; Mott and Berry 1986), a lowered cellular concentration of Mg^2+^ could be a cause for the less pronounced AO signal decrease.

## Conclusion

Mg^2+^ is the central ion of Chl molecules, and thus a significant amount of the intracellular Mg^2+^ is part of Chl molecules in cyanobacteria. Accordingly, the Chl *a* content per cell was significantly lowered when *Synechocystis* cells were grown in LM medium. This reduction in Chl led to a remarkably decreased PSI content, and related changes in electron transport properties. Additionally, Mg^2+^ limitation resulted in an altered pH gradient build up across the TM under illumination.

## Electronic supplementary material

Below is the link to the electronic supplementary material.

Supplementary Material 1

## Data Availability

No datasets were generated or analysed during the current study.
