# Supplementary material for: Mg2+ limitation leads to a decrease in chlorophyll, resulting in an unbalanced photosynthetic apparatus in the cyanobacterium Synechocytis sp. PCC6803
Source: Photosynth Res. 2024 Jul 22;162(1):13–27. doi: 10.1007/s11120-024-01112-7 (PMC11413038; doi:10.1007/s11120-024-01112-7)
Supplement: Supplementary file 1 — Supplementary Material 1 [file 11120_2024_1112_MOESM1_ESM.pdf]

Supplementary Information

Mg^{2+} limitation leads to a decrease in chlorophyll, resulting in an unbalanced photosynthetic apparatus in the cyanobacterium *Synechocytis* sp. PCC6803

Anne-Christin Pohland[1], Gábor Bernát[3], Stefan Geimer[4] and Dirk Schneider[1,2]

[1]Department of Chemistry, Biochemistry, and [2]Institute of Molecular Physiology, Johannes Gutenberg University Mainz, Germany; [3]HUN-REN Balaton Limnological Research Institute, Tihany, Hungary; [4]Cell Biology and Electron Microscopy, University of Bayreuth, Bayreuth, Germany

Table. 1: Modified BG11 used in the measurements.

**standard BG-FPC (100x)**			**BG-FPC (100x) without $MgSO_4$**		
**Substance**	**g**	**mmol/L**	**Substance**	**g**	**mmol/L**
$NaNO_3$	149.58	1.76			
Citric acid	0.66	3.44			
$CaCl_2x2H_2O$	3.6	24.49			
$MgSO_4$	7.49	30.39	Na_2SO_4	4.32	30.41
NaEDTA pH 8	1.12 **mL** (250 mM)	0.28			
Add 1 L of ultrapure H2O and filter sterile					
**Trace minerals**	**g**	**mmol/L**			
H_3BO_3	1.43	46.26			
$MnCl_2x4H_2O$	0.905	9.15			
$Na_2MoO_4x2H_2O$	0.111	0.92			
$Co(NO_3)2x6H_2O$	0.195	1.34			
$CuSO_4x5H_2O$	0.04	0.32			
Add 0.5 L of ultrapure H_2O and filter sterile					
**preparation of 1 L BG11**					
**Substance**		**mL**			
Autoclaved ultrapure H_2O		981			
BG-FPC (100x)		10			
1M HEPES-KOH pH 8.2		5			
Trace minerals		1			
6 mg/ml Ammonium ferric citrate in ultrapure H_2O		1			
190 mM Na_2CO_3 in ultrapure H_2O		1			
175 mM K_2HPO_4 in ultrapure H_2O		1			

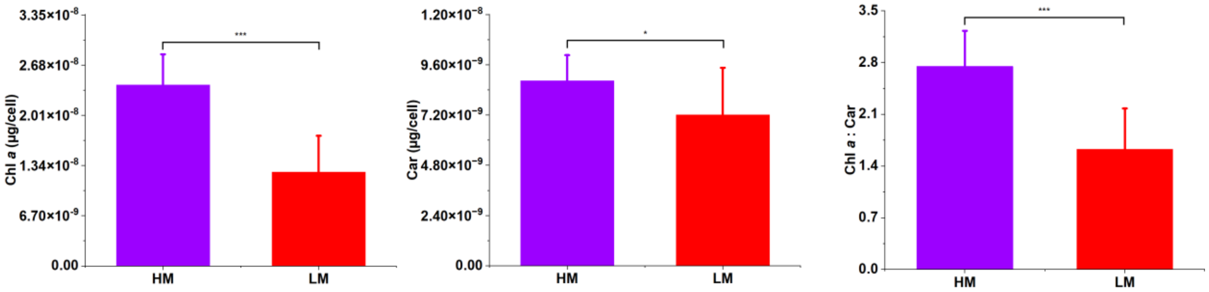

Fig. 1 The cellular Chl *a* and Car content

Cellular Chl *a* and Car contents as determined using methanolic extracts revealed a reduced amount of Chl *a* per cell (A), only minor changes in the amount of Car per cell (B), and thus a lower Chl *a*-to-Car ratio (C). Error bars represent means ± SD (N = 7–13).

Significant differences (according to Student's t-test) are indicated as: * $p < 0.05$ and *** $p < 0.001$.

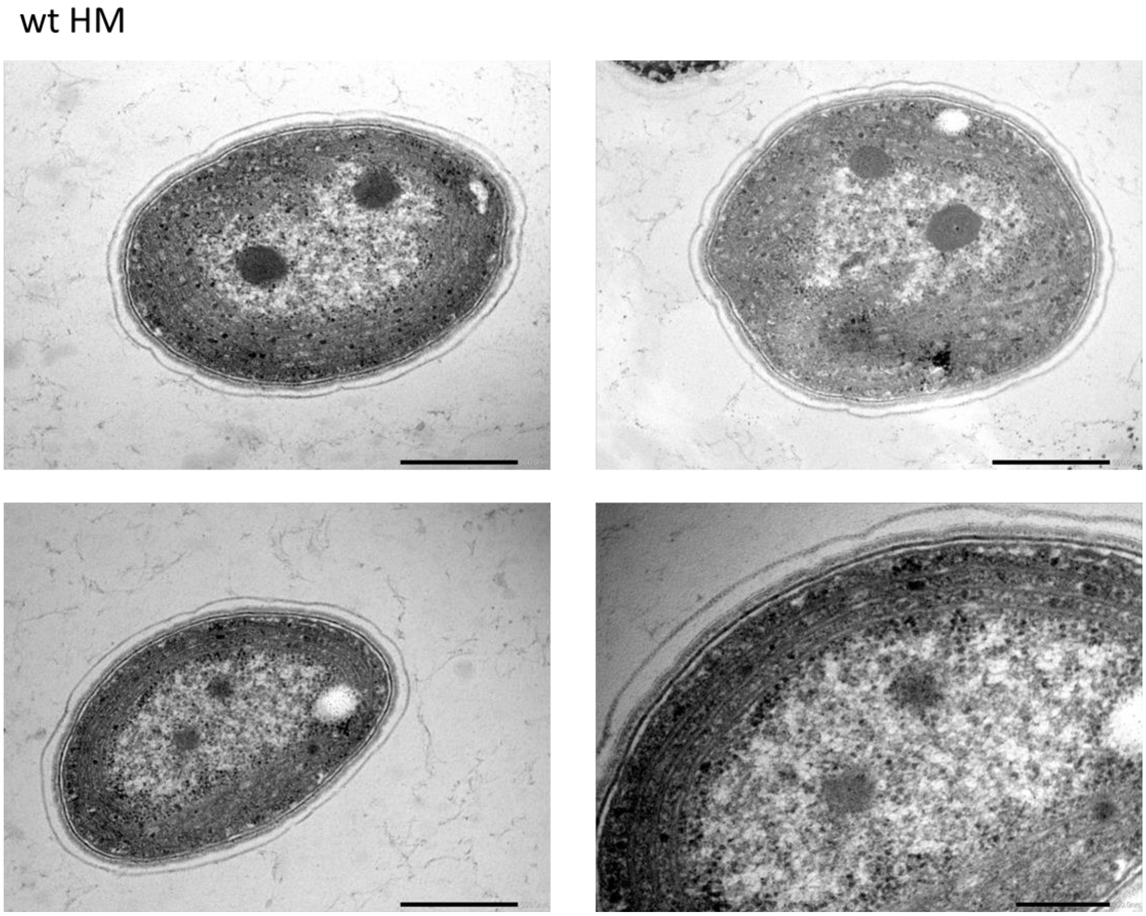

Fig. 2 EM images of *Synechocystis* cells grown under HM conditions. Scale bars = 500 nm.

wt LM

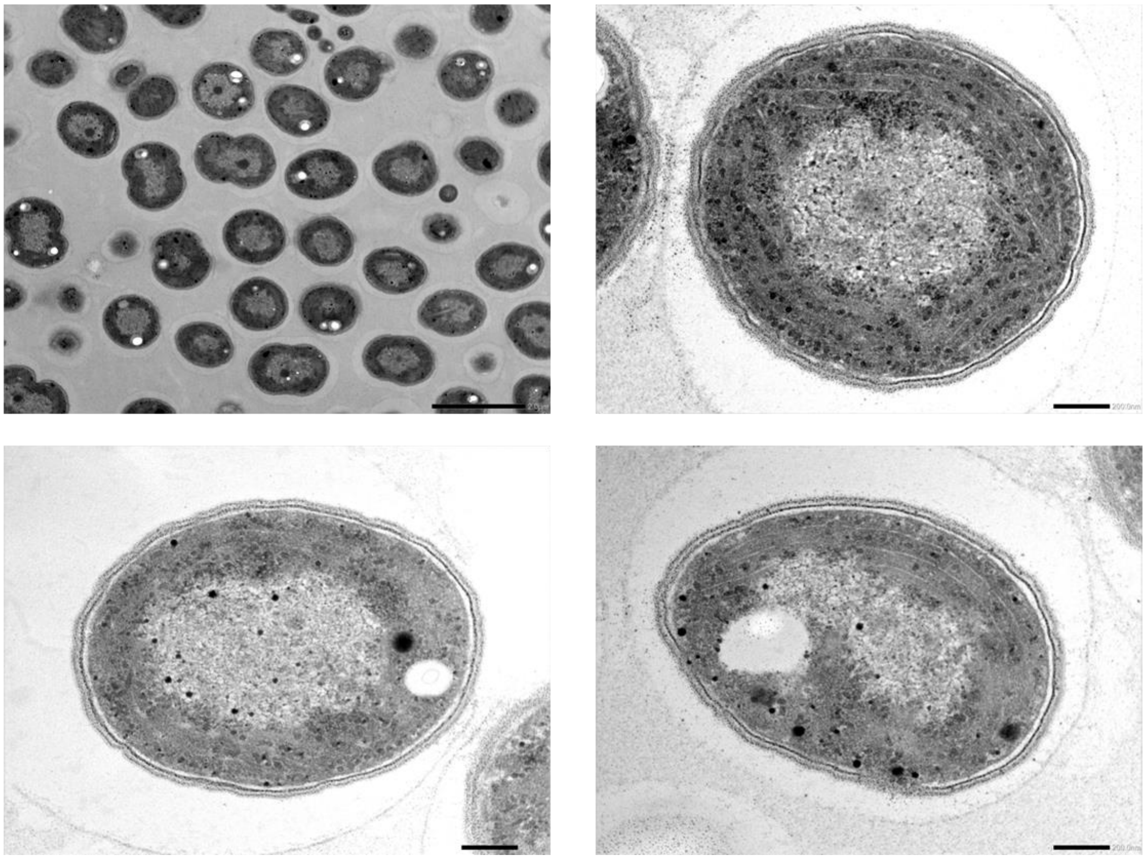

Fig. 3 EM images of *Synechocytis* cells grown under LM conditions showing an extended EPS layer
From the overview it can be seen that all cells have an extensive EPS layer. Scale bars = 200 nm, except upper left (2 μm).

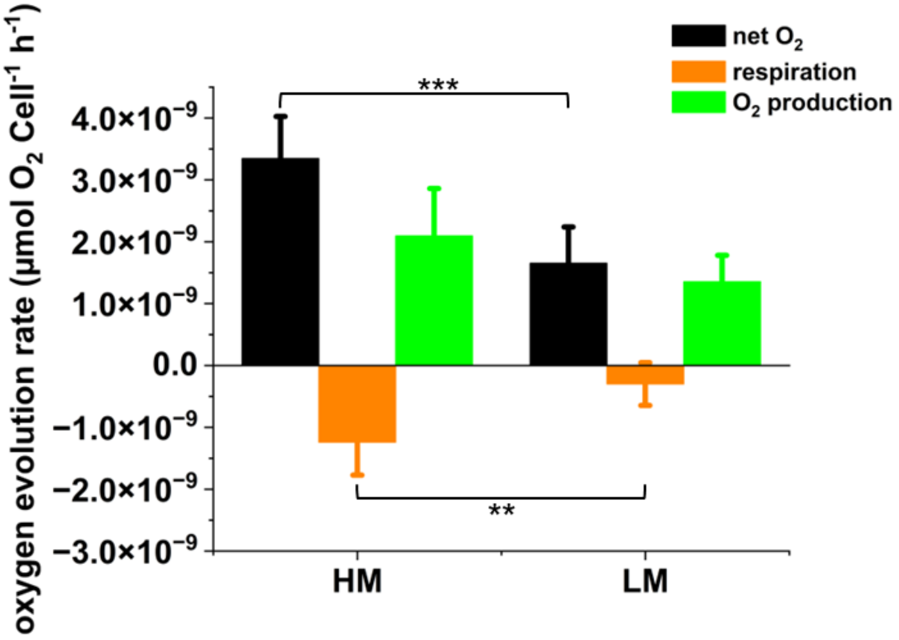

Fig. 4 O_2 consumption and evolution rates of HM and LM grown *Synechocystis*
Net O_2 production (black), O_2 consumption (orange), and O_2 evolution (green) per cell are shown for HM and LM grown cultures. Error bars represent means ± SD (N=5 (HM) or N= 7 (LM)). Significant differences (according to Student's t-test) are indicated as: ** $p < 0.01$ and *** $p < 0.001$.
